# Supplementary material for: HyperMPNN–A general strategy to design thermostable proteins learned from hyperthermophiles
Source: bioRxiv. 2024 Dec 1:2024.11.26.625397. Preprint. [Version 1] doi: 10.1101/2024.11.26.625397 (PMC11623624; doi:10.1101/2024.11.26.625397)
Supplement: Supplement 1 [file media-1.pdf]

Supplementary Information

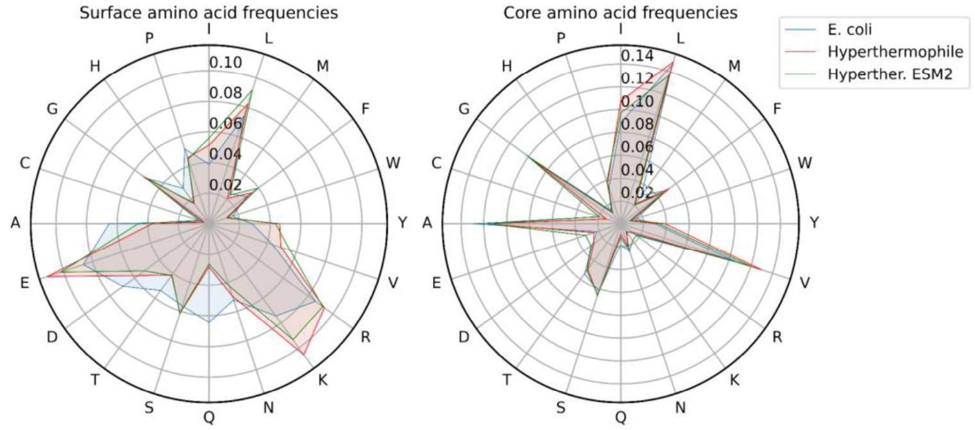

Fig. S1. Radar plot of amino acid frequencies for protein residues grouped by solvent-accessible surface area in core (<30Å) or surface (>30Å). Comparison of the frequencies for proteins from *E. coli* (blue), hyperthermophiles (red), or hyperthermophilic proteins redesigned with ESM (green).

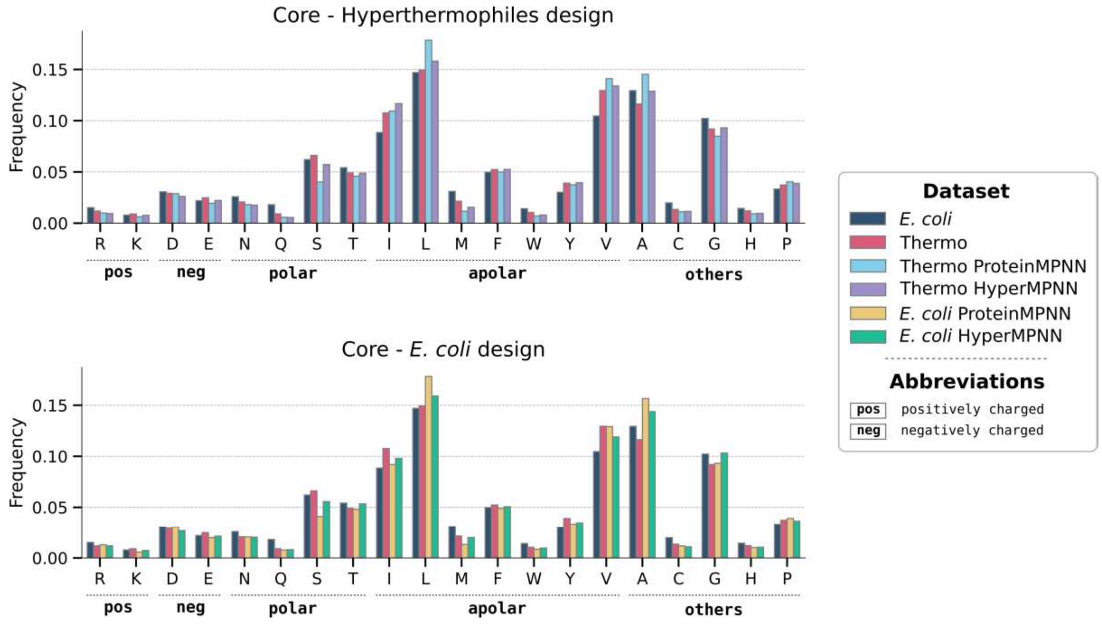

Fig. 3. ProteinMPNN fails to recover the unique amino acid composition of hyperthermophiles. Barplots of amino acid frequencies for core protein residues identified by solvent-accessible surface area (<30Å). Comparison of the frequencies for proteins from *E. coli* (dark blue), proteins from hyperthermophiles (red) . (**top**) Hyper- thermophile proteins redesigned with ProteinMPNN (light blue) and proteins from hyperthermophiles redesigned with HyperMPNN (violet). (**bottom**) *E. coli* proteins either redesigned with ProteinMPNN (yellow) or the re-trained HyperMPNN (green).

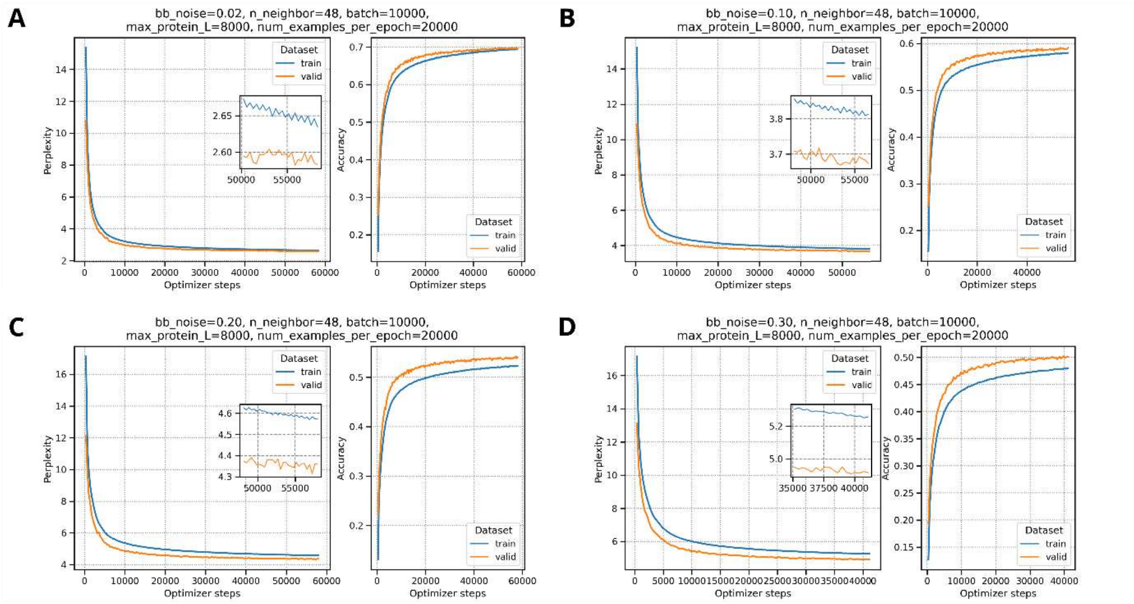

Fig. S3. Training results for HyperMPNN with different values of added backbone noise. Other parameters were held fixed for the training runs. A full list of parameters is shown in Table S2. The amount of backbone noise added during training is for (A) 0.02, (B) 0.10, (C) 0.20, and (D) 0.30.

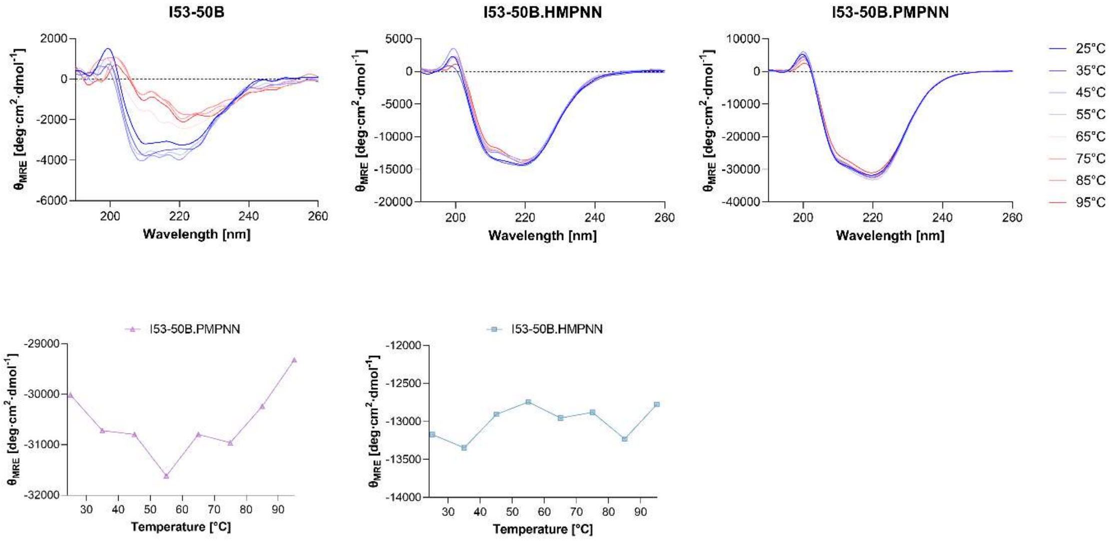

Fig. S4. Thermal melting CD spectra of I53-50B designs and parent sequence. Spectra were recorded over a temperature gradient from 25°C to 95°C. The CD signal is reported as mean residue molar ellipticity Θ_{MRE}. The bottom two diagrams illustrate the normalized signal at 223 nm of the two designs plotted against temperature, indicating no detectable thermal transition. I53-50B.hypCS represent the HyperMPNN and I5350B.protCS the ProteinMPNN designed (consensus) sequence.

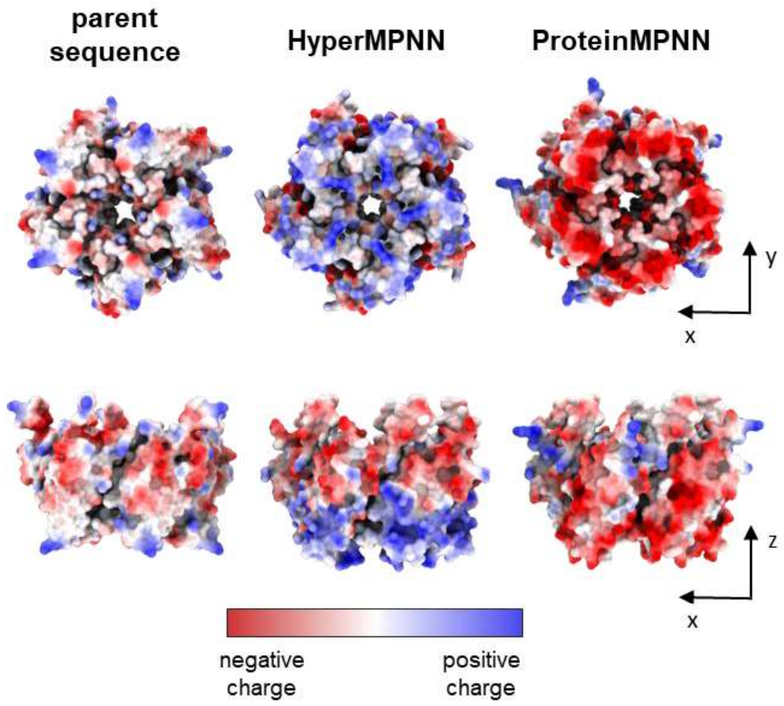

Fig. S5. Surface charge distribution of I53-50B and designs. The protein surface is color-coded according to the electrostatic potential, from negative charges (red) to positive charges (blue). The determination of charges was conducted using the Amber 20 built-in of ChimeraX.

Tab. S1. Extracted information from the pdb input structures which are mandatory for the training script to function correctly. The information is stored in pytorch-specific `pt` files (two data files per structure). Pseudo values, mandatory fields that are not used here during training, have a default value. The field with the comment containing `'pdbx_struct_assembly_gen'` is also not used because we only use single-chain pdbs.

Data files	Field	Datatype	Default	Comment
**General structure file**	seq	`list[list[str]]`	-	List of sequences for the whole structure.
	method	`str`	`'biopython'`	NMR, etc.
	date	`str`	`'2023-01-01'`	Pseudo creation date
	resolution	`float`	`0.0`	-
	chains	`list[str]`	`['A']`	All chains in structure.
	id	`str`	-	Pseudo four letter id created.
	asmb_chains	`list[str]`	`['A']`	CIF field, `pdbx_struct_assembly_gen.asym_id_list`
	asmb_details	`list[str]`	`['own_parsing']`	CIF field, `pdbx_struct_assembly_gen.details`
	asmb_method	`list[str]`	`['none']`	CIF field, `pdbx_struct_assembly_gen.method_details`

	asmd_ids	`list[str]`	`['1']`	CIF field, `pdbx_struct_assembly_gen.assembly_id`
	asmb_xform0	`tensor`	I_4, Identity matrix	-
	tm	`tensor`	`[[[1., 1., 0]]]`	Output of TMalign
**Chain specific file**	seq	`str`	-	Sequence of chain/structure
	xyz	`tensor` Shape: `Lx14x3`	-	XYZ coordinates of every atom in every sidechain, max. lengths 2. dim = 14 (Tryptophan)

Tab S2. The setting used for the training script provided in the ProteinMPNN GitHub to train HyperMPNN with structures from hyperthermophilic proteins and hyper parameter settings. Values that differ from the default settings are marked with an asterisk (*). If the parameter was changed during the hyper parameter search, the values will be listed in the last column.

Flag	Value	Hyperparameter settings
`num_epochs`	300 (*)	-
`save_model_every_n_epochs`	10	-
`reload_data_every_n_epochs`	2	-
`num_examples_per_epoch`	1000 (*)	[15000, 20000, 35000]
`batch_size`	2000 (*)	[8000, 10000, 15000]
`max_protein_length`	5000 (*)	-
`hidden_dim`	128	[96, 128, 156]
`num_encoder_layers`	3	-
`num_decoder_layers`	3	-
`num_neighbors`	48	[38, 48, 58]
`dropout`	0.1	[0.05, 0.1, 0.15]
`backbone_noise`	0.2	[0.1]
`rescut`	3.5	-
`gradient_norm`	-1.0	-
`mixed_precision`	True	-
`debug`	False	-

Tab S3. Results for the hyper parameter grid search. The values for perplexity train/valid, overfitting (bool), and the accuracy for the training and validation set correspond to the final evaluation at the end of the training loop.

batch size	Examples per epoch	Number of neighbors	Hidden Dim.	Dropout	Total time steps	perplexity train	perplexity valid	Over-fitting	accuracy train	accuracy valid
8000	15000	38	96	0.05	87386	3.907	3.768	FALSE	0.572	0.583
8000	15000	38	96	0.1	87394	4.008	3.764	FALSE	0.564	0.581
8000	15000	38	96	0.15	87390	4.083	3.763	FALSE	0.558	0.581
8000	15000	38	128	0.05	87390	3.666	3.734	TRUE	0.592	0.585
8000	15000	38	128	0.1	87392	3.768	3.668	FALSE	0.583	0.589
8000	15000	38	128	0.15	87388	3.846	3.665	FALSE	0.577	0.589
8000	15000	38	156	0.05	87392	3.481	3.764	TRUE	0.609	0.583
8000	15000	38	156	0.1	87394	3.601	3.647	TRUE	0.598	0.592
8000	15000	38	156	0.15	87396	3.692	3.607	FALSE	0.59	0.594
8000	15000	48	96	0.05	87390	3.901	3.76	FALSE	0.572	0.582
8000	15000	48	96	0.1	87390	4	3.762	FALSE	0.564	0.581
8000	15000	48	96	0.15	87394	4.072	3.786	FALSE	0.559	0.58
8000	15000	48	128	0.05	87390	3.658	3.712	TRUE	0.593	0.587
8000	15000	48	128	0.1	87394	3.761	3.688	FALSE	0.584	0.589
8000	15000	48	128	0.15	87394	3.849	3.658	FALSE	0.577	0.591
8000	15000	48	156	0.05	87396	3.478	3.737	TRUE	0.61	0.585
8000	15000	48	156	0.1	87386	3.59	3.636	TRUE	0.599	0.593
8000	15000	48	156	0.15	87394	3.687	3.595	FALSE	0.59	0.596
8000	15000	58	96	0.05	87392	3.907	3.753	FALSE	0.572	0.583
8000	15000	58	96	0.1	87392	3.994	3.74	FALSE	0.565	0.584
8000	15000	58	96	0.15	87376	4.085	3.76	FALSE	0.558	0.582
8000	15000	58	128	0.05	87396	3.653	3.709	TRUE	0.593	0.587

8000	15000	58	128	0.1	87394	3.769	3.657	FALSE	0.583	0.591
8000	15000	58	128	0.15	87392	3.845	3.646	FALSE	0.577	0.591
8000	15000	58	156	0.05	87382	3.473	3.724	TRUE	0.61	0.587
8000	15000	58	156	0.1	87390	3.601	3.656	TRUE	0.598	0.59
8000	15000	58	156	0.15	87386	3.691	3.586	FALSE	0.59	0.596
8000	20000	38	96	0.05	87392	3.909	3.768	FALSE	0.571	0.581
8000	20000	38	96	0.1	87392	4.006	3.772	FALSE	0.564	0.58
8000	20000	38	96	0.15	87394	4.078	3.749	FALSE	0.558	0.581
8000	20000	38	128	0.05	87396	3.666	3.706	TRUE	0.592	0.588
8000	20000	38	128	0.1	87394	3.769	3.652	FALSE	0.583	0.59
8000	20000	38	128	0.15	87384	3.845	3.654	FALSE	0.577	0.59
8000	20000	38	156	0.05	87394	3.483	3.75	TRUE	0.609	0.586
8000	20000	38	156	0.1	87394	3.598	3.628	TRUE	0.599	0.593
8000	20000	38	156	0.15	87398	3.688	3.608	FALSE	0.59	0.594
8000	20000	48	96	0.05	87390	3.904	3.76	FALSE	0.572	0.582
8000	20000	48	96	0.1	87384	3.995	3.73	FALSE	0.565	0.584
8000	20000	48	96	0.15	87392	4.079	3.737	FALSE	0.558	0.583
8000	20000	48	128	0.05	87390	3.65	3.711	TRUE	0.594	0.587
8000	20000	48	128	0.1	87392	3.759	3.644	FALSE	0.584	0.591
8000	20000	48	128	0.15	87394	3.845	3.645	FALSE	0.577	0.593
8000	20000	48	156	0.05	87390	3.475	3.749	TRUE	0.61	0.585
8000	20000	48	156	0.1	87388	3.598	3.648	TRUE	0.598	0.592
8000	20000	48	156	0.15	87400	3.69	3.59	FALSE	0.59	0.595
8000	20000	58	96	0.05	87394	3.909	3.749	FALSE	0.572	0.584
8000	20000	58	96	0.1	87386	4.003	3.761	FALSE	0.564	0.583
8000	20000	58	96	0.15	87392	4.074	3.745	FALSE	0.559	0.583
8000	20000	58	128	0.05	87388	3.653	3.709	TRUE	0.593	0.588
8000	20000	58	128	0.15	87384	3.845	3.641	FALSE	0.577	0.593
8000	20000	58	156	0.05	87394	3.477	3.727	TRUE	0.609	0.587
8000	20000	58	156	0.1	87394	3.587	3.628	TRUE	0.599	0.593
8000	20000	58	156	0.15	87382	3.687	3.592	FALSE	0.591	0.596
8000	35000	38	96	0.05	87388	3.917	3.776	FALSE	0.571	0.581
8000	35000	38	96	0.1	87388	4.008	3.754	FALSE	0.564	0.582
8000	35000	38	96	0.15	87396	4.085	3.764	FALSE	0.558	0.581
8000	35000	38	128	0.05	87390	3.666	3.718	TRUE	0.592	0.586
8000	35000	38	128	0.1	87392	3.771	3.653	FALSE	0.583	0.591
8000	35000	38	128	0.15	87390	3.853	3.645	FALSE	0.576	0.592
8000	35000	38	156	0.05	87388	3.483	3.724	TRUE	0.609	0.587
8000	35000	38	156	0.1	87394	3.592	3.662	TRUE	0.599	0.59
8000	35000	38	156	0.15	87388	3.687	3.605	FALSE	0.59	0.595
8000	35000	48	96	0.05	87394	3.899	3.747	FALSE	0.573	0.585
8000	35000	48	96	0.1	87394	3.999	3.732	FALSE	0.564	0.585
8000	35000	48	96	0.15	87392	4.081	3.763	FALSE	0.558	0.582
8000	35000	48	128	0.05	87390	3.65	3.691	TRUE	0.593	0.589
8000	35000	48	128	0.1	87390	3.767	3.661	FALSE	0.583	0.592
8000	35000	48	128	0.15	87398	3.843	3.64	FALSE	0.577	0.592
8000	35000	48	156	0.05	87392	3.483	3.731	TRUE	0.609	0.586
8000	35000	48	156	0.1	87384	3.597	3.628	TRUE	0.598	0.592
8000	35000	48	156	0.15	87398	3.685	3.594	FALSE	0.59	0.597
8000	35000	58	96	0.05	87390	3.895	3.733	FALSE	0.573	0.584
8000	35000	58	96	0.1	87392	3.991	3.724	FALSE	0.565	0.586
8000	35000	58	96	0.15	87392	4.086	3.734	FALSE	0.558	0.585
8000	35000	58	128	0.05	87392	3.649	3.689	TRUE	0.594	0.59
8000	35000	58	128	0.1	87390	3.76	3.649	FALSE	0.584	0.591
8000	35000	58	128	0.15	87396	3.847	3.643	FALSE	0.577	0.591
8000	35000	58	156	0.05	87396	3.475	3.724	TRUE	0.61	0.587
8000	35000	58	156	0.1	87390	3.584	3.635	TRUE	0.6	0.593
8000	35000	58	156	0.15	87394	3.685	3.595	FALSE	0.591	0.596
10000	15000	38	96	0.05	70266	3.92	3.785	FALSE	0.571	0.58
10000	15000	38	96	0.1	70266	4.011	3.763	FALSE	0.563	0.582
10000	15000	38	96	0.15	70290	4.091	3.763	FALSE	0.557	0.581
10000	15000	38	128	0.05	70276	3.666	3.726	TRUE	0.592	0.587
10000	15000	38	128	0.1	70256	3.766	3.67	FALSE	0.583	0.59
10000	15000	38	128	0.15	70260	3.839	3.642	FALSE	0.577	0.592
10000	15000	38	156	0.05	70264	3.47	3.751	TRUE	0.61	0.585
10000	15000	38	156	0.1	70250	3.593	3.664	TRUE	0.599	0.59
10000	15000	38	156	0.15	70260	3.682	3.605	FALSE	0.591	0.594
10000	15000	48	96	0.05	70266	3.903	3.775	FALSE	0.572	0.581
10000	15000	48	96	0.1	70272	4.005	3.762	FALSE	0.564	0.582
10000	15000	48	96	0.15	70266	4.085	3.75	FALSE	0.558	0.584
10000	15000	48	128	0.05	70284	3.653	3.713	TRUE	0.594	0.587
10000	15000	48	128	0.1	70276	3.757	3.662	FALSE	0.585	0.591
10000	15000	48	128	0.15	70258	3.835	3.63	FALSE	0.578	0.592
10000	15000	48	156	0.05	70266	3.458	3.75	TRUE	0.611	0.585
10000	15000	48	156	0.1	70258	3.59	3.656	TRUE	0.599	0.592
10000	15000	48	156	0.15	70264	3.679	3.609	FALSE	0.591	0.595
10000	15000	58	96	0.05	70282	3.899	3.783	FALSE	0.573	0.581
10000	15000	58	96	0.1	70260	3.996	3.746	FALSE	0.565	0.584
10000	15000	58	96	0.15	70264	4.079	3.755	FALSE	0.558	0.583
10000	15000	58	128	0.05	70260	3.646	3.719	TRUE	0.594	0.587
10000	15000	58	128	0.1	70274	3.76	3.659	FALSE	0.584	0.592
10000	15000	58	128	0.15	70260	3.843	3.635	FALSE	0.577	0.593

10000	15000	58	156	0.05	70278	3.47	3.73	TRUE	0.61	0.586
10000	15000	58	156	0.1	70264	3.587	3.66	TRUE	0.599	0.59
10000	15000	58	156	0.15	70256	3.679	3.586	FALSE	0.591	0.597
10000	20000	38	96	0.05	70284	3.913	3.761	FALSE	0.572	0.583
10000	20000	38	96	0.1	70260	4.008	3.746	FALSE	0.564	0.584
10000	20000	38	96	0.15	70280	4.102	3.759	FALSE	0.557	0.583
10000	20000	38	128	0.05	70274	3.652	3.728	TRUE	0.594	0.585
10000	20000	38	128	0.1	70280	3.76	3.663	FALSE	0.584	0.59
10000	20000	38	128	0.15	70264	3.85	3.652	FALSE	0.577	0.591
10000	20000	38	156	0.05	70256	3.469	3.745	TRUE	0.61	0.585
10000	20000	38	156	0.1	70262	3.592	3.666	TRUE	0.599	0.59
10000	20000	38	156	0.15	70268	3.688	3.62	FALSE	0.59	0.594
10000	20000	48	96	0.05	70266	3.903	3.756	FALSE	0.572	0.584
10000	20000	48	96	0.1	70266	3.994	3.739	FALSE	0.565	0.584
10000	20000	48	96	0.15	70272	4.078	3.761	FALSE	0.559	0.584
10000	20000	48	128	0.05	70270	3.663	3.724	TRUE	0.592	0.586
10000	20000	48	128	0.1	70262	3.759	3.658	FALSE	0.584	0.591
10000	20000	48	128	0.15	70266	3.846	3.632	FALSE	0.577	0.593
10000	20000	48	156	0.05	70270	3.458	3.745	TRUE	0.612	0.586
10000	20000	48	156	0.1	70266	3.588	3.655	TRUE	0.599	0.593
10000	20000	48	156	0.15	70278	3.682	3.614	FALSE	0.591	0.595
10000	20000	58	96	0.05	70252	3.901	3.751	FALSE	0.572	0.583
10000	20000	58	96	0.1	70278	4.005	3.738	FALSE	0.564	0.585
10000	20000	58	96	0.15	70262	4.085	3.737	FALSE	0.558	0.583
10000	20000	58	128	0.05	70272	3.656	3.706	TRUE	0.593	0.588
10000	20000	58	128	0.1	70288	3.752	3.656	FALSE	0.585	0.591
10000	20000	58	128	0.15	70268	3.841	3.617	FALSE	0.577	0.593
10000	20000	58	156	0.05	70260	3.46	3.733	TRUE	0.611	0.587
10000	20000	58	156	0.1	70284	3.579	3.67	TRUE	0.6	0.59
10000	20000	58	156	0.15	70274	3.679	3.604	FALSE	0.591	0.595
10000	35000	38	96	0.05	70264	3.913	3.775	FALSE	0.572	0.583
10000	35000	38	96	0.1	70274	4.007	3.754	FALSE	0.564	0.583
10000	35000	38	96	0.15	70280	4.087	3.768	FALSE	0.558	0.581
10000	35000	38	128	0.05	70278	3.664	3.725	TRUE	0.593	0.587
10000	35000	38	128	0.1	70266	3.759	3.669	FALSE	0.584	0.589
10000	35000	38	128	0.15	70270	3.847	3.662	FALSE	0.577	0.589
10000	35000	38	156	0.05	70284	3.47	3.757	TRUE	0.61	0.585
10000	35000	38	156	0.1	70258	3.598	3.663	TRUE	0.598	0.59
10000	35000	38	156	0.15	70262	3.683	3.61	FALSE	0.591	0.596
10000	35000	48	96	0.05	70258	3.906	3.76	FALSE	0.572	0.584
10000	35000	48	96	0.1	70290	3.998	3.721	FALSE	0.564	0.586
10000	35000	48	96	0.15	70252	4.092	3.77	FALSE	0.557	0.582
10000	35000	48	128	0.05	70270	3.652	3.713	TRUE	0.594	0.588
10000	35000	48	128	0.1	70284	3.757	3.644	FALSE	0.584	0.591
10000	35000	48	128	0.15	70288	3.839	3.643	FALSE	0.578	0.592
10000	35000	48	156	0.05	70276	3.466	3.749	TRUE	0.611	0.586
10000	35000	48	156	0.1	70278	3.584	3.645	TRUE	0.599	0.592
10000	35000	48	156	0.15	70264	3.681	3.603	FALSE	0.591	0.596
10000	35000	58	96	0.05	70290	3.908	3.77	FALSE	0.572	0.583
10000	35000	58	96	0.1	70268	4.004	3.745	FALSE	0.564	0.582
10000	35000	58	96	0.15	70270	4.086	3.776	FALSE	0.558	0.581
10000	35000	58	128	0.05	70282	3.657	3.714	TRUE	0.593	0.587
10000	35000	58	128	0.1	70284	3.756	3.658	FALSE	0.584	0.592
10000	35000	58	128	0.15	70274	3.836	3.632	FALSE	0.578	0.593
10000	35000	58	156	0.05	70272	3.47	3.74	TRUE	0.61	0.586
10000	35000	58	156	0.1	70288	3.581	3.654	TRUE	0.6	0.591
10000	35000	58	156	0.15	70274	3.678	3.611	FALSE	0.591	0.594
15000	15000	38	96	0.05	47302	3.935	3.806	FALSE	0.569	0.579
15000	15000	38	96	0.1	47316	4.027	3.768	FALSE	0.562	0.582
15000	15000	38	96	0.15	47312	4.113	3.773	FALSE	0.556	0.58
15000	15000	38	128	0.05	47318	3.67	3.746	TRUE	0.592	0.585
15000	15000	38	128	0.1	47322	3.776	3.669	FALSE	0.583	0.59
15000	15000	38	128	0.15	47296	3.861	3.664	FALSE	0.576	0.59
15000	15000	38	156	0.05	47302	3.474	3.816	TRUE	0.61	0.58
15000	15000	38	156	0.1	47296	3.589	3.696	TRUE	0.599	0.588
15000	15000	38	156	0.15	47302	3.691	3.626	FALSE	0.59	0.594
15000	15000	48	96	0.05	47304	3.924	3.785	FALSE	0.571	0.581
15000	15000	48	96	0.1	47308	4.01	3.739	FALSE	0.563	0.584
15000	15000	48	96	0.15	47318	4.086	3.755	FALSE	0.558	0.582
15000	15000	48	128	0.05	47318	3.663	3.736	TRUE	0.593	0.585
15000	15000	48	128	0.1	47308	3.766	3.669	FALSE	0.583	0.59
15000	15000	48	128	0.15	47302	3.839	3.63	FALSE	0.578	0.592
15000	15000	48	156	0.05	47300	3.464	3.814	TRUE	0.611	0.578
15000	15000	48	156	0.1	47300	3.588	3.66	TRUE	0.599	0.591
15000	15000	48	156	0.15	47314	3.681	3.613	FALSE	0.591	0.594
15000	15000	58	96	0.05	47302	3.918	3.763	FALSE	0.571	0.583
15000	15000	58	96	0.1	47292	4.012	3.777	FALSE	0.564	0.583
15000	15000	58	96	0.15	47316	4.095	3.752	FALSE	0.557	0.582
15000	15000	58	128	0.05	47312	3.656	3.727	TRUE	0.593	0.586
15000	15000	58	128	0.1	47306	3.768	3.681	FALSE	0.583	0.589
15000	15000	58	128	0.15	47302	3.845	3.66	FALSE	0.577	0.59
15000	15000	58	156	0.15	47306	3.677	3.603	FALSE	0.592	0.595

15000	20000	38	96	0.05	47298	3.921	3.787	FALSE	0.571	0.581
15000	20000	38	96	0.1	47308	4.032	3.762	FALSE	0.562	0.582
15000	20000	38	96	0.15	47300	4.106	3.763	FALSE	0.556	0.58
15000	20000	38	128	0.05	47292	3.668	3.737	TRUE	0.592	0.585
15000	20000	38	128	0.1	47306	3.775	3.679	FALSE	0.583	0.589
15000	20000	38	128	0.15	47280	3.855	3.652	FALSE	0.576	0.591
15000	20000	38	156	0.05	47316	3.463	3.769	TRUE	0.611	0.582
15000	20000	38	156	0.1	47294	3.598	3.705	TRUE	0.599	0.587
15000	20000	38	156	0.15	47296	3.681	3.635	FALSE	0.591	0.591
15000	20000	48	96	0.05	47318	3.922	3.812	FALSE	0.571	0.58
15000	20000	48	96	0.1	47310	4.018	3.772	FALSE	0.563	0.583
15000	20000	48	96	0.15	47284	4.093	3.762	FALSE	0.557	0.581
15000	20000	48	128	0.05	47286	3.67	3.749	TRUE	0.592	0.583
15000	20000	48	128	0.1	47312	3.773	3.682	FALSE	0.583	0.588
15000	20000	48	128	0.15	47306	3.851	3.652	FALSE	0.576	0.592
15000	20000	48	156	0.05	47308	3.461	3.788	TRUE	0.611	0.581
15000	20000	48	156	0.1	47292	3.577	3.672	TRUE	0.6	0.589
15000	20000	48	156	0.15	47298	3.674	3.609	FALSE	0.591	0.594
15000	20000	58	96	0.05	47304	3.918	3.766	FALSE	0.571	0.583
15000	20000	58	96	0.1	47312	4.012	3.747	FALSE	0.563	0.584
15000	20000	58	96	0.15	47310	4.099	3.752	FALSE	0.557	0.583
15000	20000	58	128	0.05	47306	3.656	3.758	TRUE	0.593	0.583
15000	20000	58	128	0.1	47312	3.768	3.669	FALSE	0.584	0.59
15000	20000	58	128	0.15	47302	3.846	3.659	FALSE	0.577	0.589
15000	20000	58	156	0.05	47288	3.464	3.784	TRUE	0.611	0.582
15000	35000	38	96	0.05	47322	3.931	3.799	FALSE	0.57	0.581
15000	35000	38	96	0.1	47312	4.018	3.769	FALSE	0.563	0.582
15000	35000	38	96	0.15	47302	4.104	3.791	FALSE	0.556	0.579
15000	35000	38	128	0.05	47294	3.673	3.744	TRUE	0.592	0.584
15000	35000	38	128	0.1	47304	3.773	3.701	FALSE	0.583	0.587
15000	35000	38	128	0.15	47314	3.858	3.671	FALSE	0.576	0.588
15000	35000	38	156	0.05	47292	3.47	3.772	TRUE	0.61	0.584
15000	35000	38	156	0.1	47304	3.6	3.686	TRUE	0.598	0.589
15000	35000	38	156	0.15	47298	3.689	3.613	FALSE	0.59	0.594
15000	35000	48	96	0.05	47292	3.923	3.785	FALSE	0.57	0.581
15000	35000	48	96	0.1	47310	4.011	3.741	FALSE	0.564	0.583
15000	35000	48	96	0.15	47292	4.097	3.743	FALSE	0.557	0.584
15000	35000	48	128	0.05	47282	3.659	3.751	TRUE	0.593	0.584
15000	35000	48	128	0.1	47296	3.766	3.665	FALSE	0.584	0.591
15000	35000	48	128	0.15	47304	3.845	3.649	FALSE	0.577	0.591
15000	35000	48	156	0.05	47314	3.469	3.785	TRUE	0.611	0.583
15000	35000	48	156	0.1	47304	3.588	3.661	TRUE	0.599	0.592
15000	35000	48	156	0.15	47302	3.685	3.631	FALSE	0.59	0.592
15000	35000	58	96	0.05	47296	3.914	3.789	FALSE	0.571	0.581
15000	35000	58	96	0.1	47302	4.011	3.765	FALSE	0.563	0.582
15000	35000	58	96	0.15	47310	4.094	3.742	FALSE	0.557	0.583
15000	35000	58	128	0.05	47308	3.658	3.74	TRUE	0.593	0.586
15000	35000	58	128	0.1	47298	3.763	3.699	FALSE	0.584	0.588
15000	35000	58	128	0.15	47302	3.847	3.637	FALSE	0.577	0.592
15000	35000	58	156	0.15	32636	3.772	3.66	FALSE	0.583	0.59

584
